# Lipidation of small GTPase Cdc42 as regulator of its physiological and pathophysiological functions

**DOI:** 10.3389/fphys.2022.1088840

**Published:** 2023-01-09

**Authors:** Alexander Wirth, Evgeni Ponimaskin

**Affiliations:** Cellular Neurophysiology, Hannover Medical School, Hannover, Germany

**Keywords:** Cdc42, small GTPase, palmitoylation, prenylation, lipidation

## Abstract

The protein cell division cycle 42 (Cdc42) is a small GTPase of the Rho family regulating a plethora of physiological functions in a tissue, cell and subcellular-specific manner *via* participating in multiple signaling pathways. Since the corresponding signaling hubs are mainly organized along the cellular membranes, cytosolic proteins like Cdc42 need to be properly targeted and held at the membrane. Here, lipid modifications come into play: Cdc42 can be associated with membranes by different lipid anchors including prenylation (Cdc42-prenyl) and palmitoylation (Cdc42-palm). While Cdc42-prenyl is ubiquitously expressed, Cdc42-palm splicing variant in mainly expressed in the brain. Mechanisms underlying Cdc42 lipidation as well as its regulation are the main topic of this review. Furthermore, we will discuss the functional importance of Cdc42 lipid modifications with the focus on the role of different lipids in regulating defined Cdc42 functions. Finally, we will provide an overview of the possible implementation of Cdc42 lipidation in pathological conditions and different diseases.

## Structure and distribution of Cdc42 splicing variants

The protein cell division cycle 42 (Cdc42) was initially purified from the placental membranes during isolation of small molecular weight GTP hydrolyzing proteins (Evans et al., 1986). This protein was termed G_p_ (G_p_ = placental G protein). When later researchers investigated the interplay between cell cycle events (e.g., DNA replication) and cytosolic/membrane events (e.g., budding) in yeast *Saccharomyces cerevisiae*, they identified a mutant that was able to continue DNA synthesis and nuclear division while the emergence of buds was blocked. The responsible gene was named CDC42, in relation to another budding-involved gene, namely CDC24 (Adams et al., 1990). In the same year, Shinjo et al. isolated the cDNA clone for G_p_ (or G25K) and found that it has the highest degree of sequence identity (more than 80%) with the CDC42. Therefore, the corresponding human placental protein was designated Cdc42Hs. Using different probes, authors not only isolated cDNA for the Cdc42Hs, but also found a second protein with the same size but different C-terminal sequence (G_p_’), concluding that these might be another reading frame within the same gene (Shinjo et al., 1990).

**FIGURE 1:**
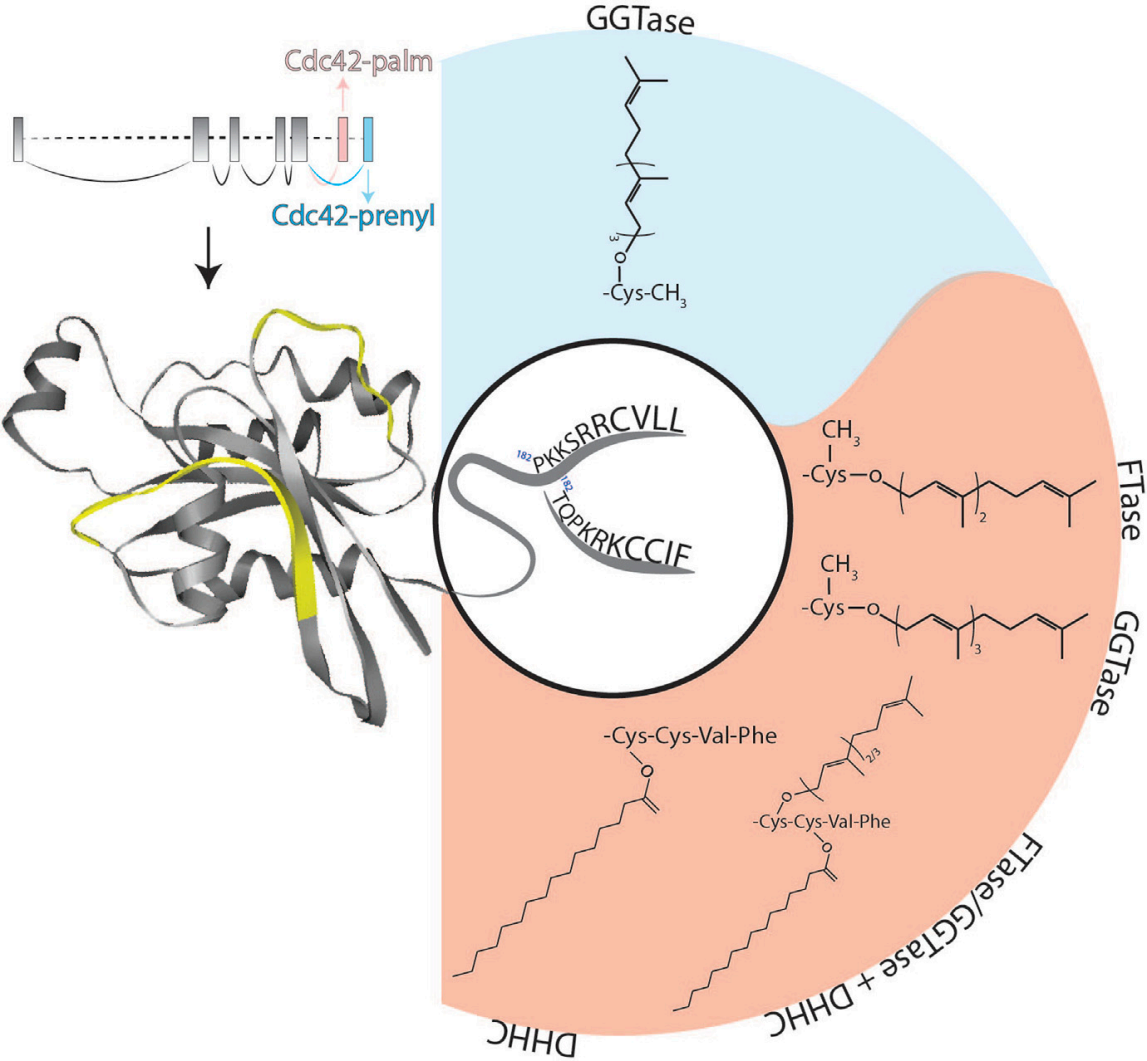
Schematic representation of the structure of Cdc42 alternatively spliced exons as well as protein and its lipid modifications. Two flexible switch regions are highlighted in yellow. The two different C-termini of Cdc42-palm (red background) and Cdc42-prenyl (blue background) isoforms together with their possible post-translational lipid modifications are shown. DHHC, palmitoyl acyl-transferase harboring a cysteine-rich domain containing a conserved DHHC (Asp-His-His-Cys) motif; FTase, farnesyltransferase; GGTase, geranylgeranyltransferase.

Today, we know that these two cDNAs refer to two alternatively spliced isoforms of Cdc42 mapping to chromosome 1p36.1 in humans (Nicole et al., 1999) and to chromosome 4 in mice (Marks and Kwiatkowski, 1996). The first isoform is also referred as G25K, Gp, Cdc42a, Cdc42-prenyl, Cdc42E7, Cdc42u or Cdc42-v1, while the second—as G_p_’, Cdc42-palm, Cdc42E6, Cdc42b, bCdc42, Cdc42-v2. To simplify the reading, we will mark the first isoform as Cdc42-prenyl and the second one as Cdc42-palm in the further text. The CDC42 gene consists of 7 exons, and the last two exons can be alternatively spliced (Figure 1; Marks and Kwiatkowski, 1996; Nicole et al., 1999; Yap et al., 2016). The common Cdc42 structure consists of five α-helices, six β-strands and two highly mobile switch regions with the switch I region resided between α1 and β2 loops, and switch II region within β3 and β4 loops (Figure 1). These regions are important for the substrate recognition and interaction together with the P-loop, which is also involved in binding a phosphate group of guanine nucleotides. Even though the structure of GTP and GDP-bound Cdc42 does not significantly differ, effector proteins are still able to bind to the activated form, inducing conformational changes within switch I and switch II loops, leading to initiation of the downstream signaling (Phillips et al., 2008).

**FIGURE 2:**
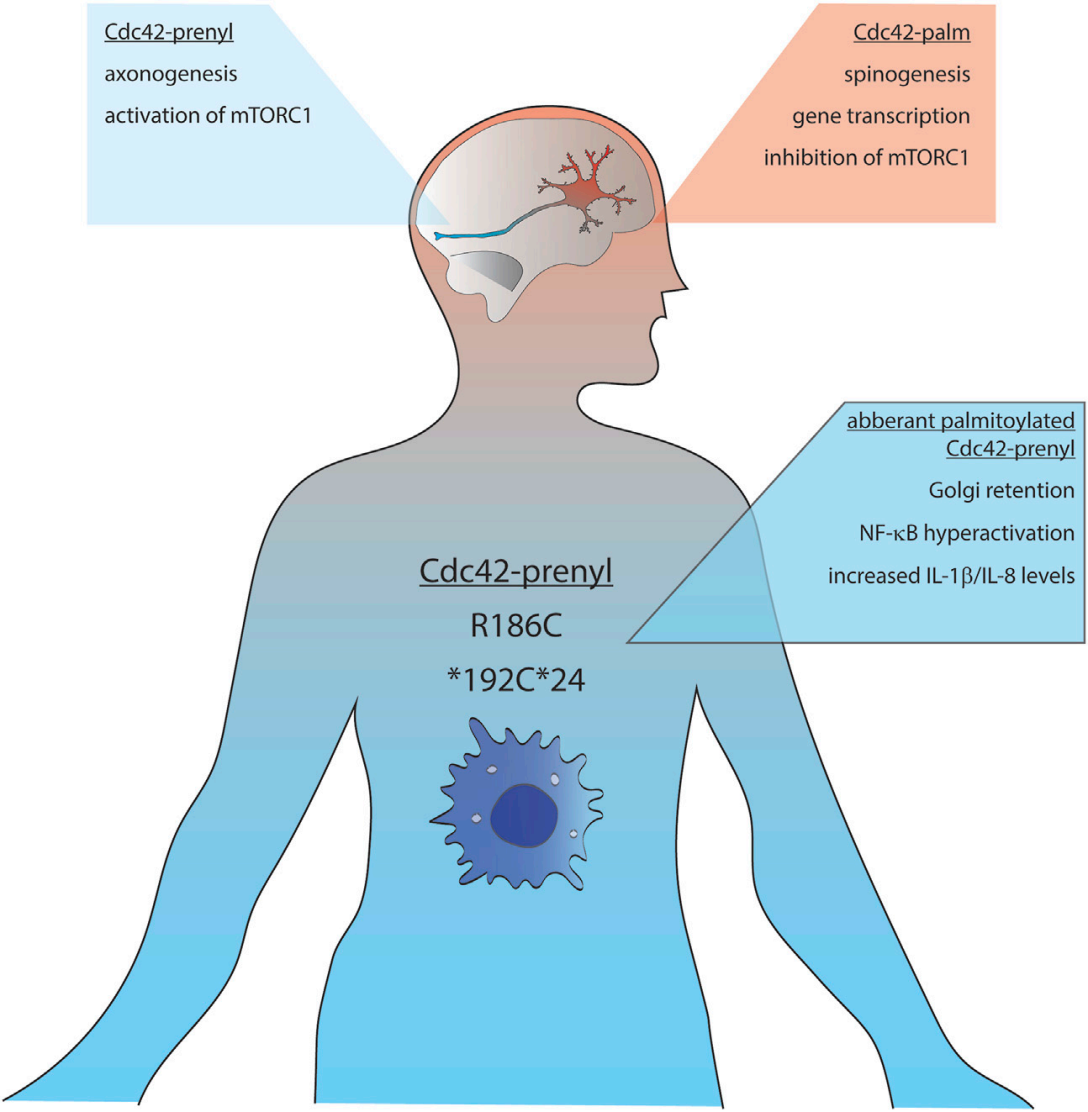
Schematic representation of the distribution and functions of Cdc42-palm (red background) and Cdc42-prenyl (blue background) isoforms. mTORC1, mammalian target of rapamycin complex 1; IL, interleukin; NF-κB, nuclear factor kappa-light-chain-enhancer of activated B cells.

Initial analysis performed by Marks et al. revealed that Cdc42-prenyl is broadly distributed within multiple tissues including uterus, thymus, gut, kidney and lung, whereas Cdc42-palm expression was restricted to the brain, where this isoform was expressed at relative low levels (Figure 2; Marks and Kwiatkowski, 1996). In the ensuing study, Olenik and colleagues investigated the expression of both Cdc42 isoforms in developing rat neocortices. They have shown that Cdc42-prenyl localizes to the proliferation zone, whereas Cdc42-palm was restricted to the cortical zone—a place in which neurons differentiate and settle, but do not proliferate anymore (Olenik et al., 1999). A recent study by Yap et al. shed light on the regulatory mechanisms of the alternative splicing of the CDC42 gene transcripts in developing embryonic stem cells, differentiating into glutamatergic neurons as well as into hippocampal neurons (Yap et al., 2016). They reported about a polypyrimidine tract-binding protein 1 (Ptbp)-dependent splicing switch, leading to the production of equal amounts of Cdc42-palm Cdc42-prenyl transcripts from day *in vitro* 7 onwards in neurons, but not astrocytes. A more recent study by Ciolli Mattioli and colleagues demonstrated that Cdc42-prenyl mRNA was enriched in neurites of mESC-derived and primary cortical neurons. In contrast, mRNA transcripts of Cdc42-palm isoform were predominantly localized to the cell somata (Ciolli Mattioli et al., 2019). Such compartmentalization of Cdc42-prenyl and Cdc42-palm splice variants seem to be preserved also within the peripheral nervous system. Indeed, Cdc42-prenyl mRNA has been shown to be specifically enriched in axons of sensory neurons of the dorsal root ganglion (Lee et al., 2021). In contrast, Kang and colleagues reported a dendritic localization of both isoforms in cultured hippocampal neurons (Kang et al., 2008). They also showed that GFP-tagged Cdc42-prenyl is distributed throughout the whole dendrite, including spines and shaft-like structures, whereas Cdc42-palm was restricted to dendritic spines.

Of note, our understanding of the physiological functions of both Cdc42 isoforms is still incomplete. The full deletion of Cdc42 in germline is embryonically lethal at E6.5, which points to its biological relevance (Chen et al., 2000). A conditional deletion of Cdc42 in the developing cortex of mice at E9.5 causes mislocalization of adherens junctions due to missing cellular polarization, usually maintained by Cdc42-Par complex interaction (Cappello et al., 2006) and a telencephalon-specific knockout leads to holoprosencephaly—a incomplete bifurcation of cerebral hemispheres (Chen et al., 2006).

## Cdc42 splicing variants: what is different?

At the protein level, Cdc42 isoforms differ in their C-terminal amino acid sequences: Cdc42-palm possesses a non-canonical *CCaX* motif (CCIF; in which C is cysteine, a—preferably aliphatic amino acid and X is any amino acid), whereas Cdc42-prenyl is terminated by a *CaaX*-box (CVLL) (Shinjo et al., 1990). In addition, alternative splicing of the exons 6 and 7 results in an altered polybasic region (PBR) and an exchange of lysine to arginine at position 163 (Figure 1). Whereas Cdc42-prenyl bears a di-lysine di-arginine motif separated by a serine (-KKSRR-), Cdc42-palm’s PBR consists only of a lysine-arginine-lysine (-KRK-) motif (Figure 1; Marks and Kwiatkowski, 1996). The polybasic region is involved in a variety of biological processes, especially in protein-protein interaction. Moreover, PBR-motifs seem to play a crucial role for the membrane sorting, including localization at different membrane compartments (e.g., plasma membrane and endo-membranes) (reviewed in Williams, 2003).

The di-lysine motif (Lys-183 and Lys-184) within Cdc42-prenyl has been shown to be involved in interaction of Cdc42-prenyl with the γCOP subunit of the coat proteins I (COPI) complex, playing a role in regulating intracellular trafficking (Wu et al., 2000). The di-arginine motif of Cdc42-prenyl (i.e., Arg-186 and Arg-187) was shown to be responsible for its association with phosphatidylinositol 4,5-bisphosphate, which correlates with the ability of this isoform to induce oncogenic transformation (Johnson et al., 2012). In contrast, the polybasic sequence Lys-185, Arg-186, Lys-187 within Cdc42-palm (Figure 1) leads to localization of this isoform into phosphatidylinositol (3,4,5)-triphosphate microdomains (Endo and Cerione, 2022), where it can act as a tumor suppressor, at least in ovarian cancer cells (He et al., 2015). Another consequence of the alternative splicing of the CDC42 gene transcripts is the presence of a canonical nuclear localization sequence KKSR exclusively within the Cdc42-prenyl isoform (Williams, 2003).

## Cdc42 prenylation

Based on sequence homology of the C-terminus of Cdc42 and Ras proteins, Johnson und Pringle predicted that Cdc42-prenyl might undergo posttranslational prenylation (Johnson and Pringle, 1990). Protein prenylation is an irreversible post-translational modification, which is involved in the regulation of localization, functions, and protein-protein interactions of multiple small GTPases, including the Ras superfamily (Zhang and Casey, 1996). Prenylation implies covalent attachment of either a farnesyl (15 carbon atoms) or a geranylgeranyl (20 carbon atoms) isoprenoid group catalyzed by different prenyltransferases, including farnesyltransferase (FTase), geranylgeranyltransferase type I (GGTase-I), Rab geranylgeranyltransferase (GGTase-II), and geranylgeranyltransferase type III (GGTase-III) (Marchwicka et al., 2022). The isoprene derivative is linked to the cysteine residue of the C-terminal CaaX-box *via* irreversible thioether bond (Casey and Seabra, 1996). In general, prenylated proteins are further processed at the cytoplasmic face of the endoplasmic reticulum (ER) by a two-step process: 1) Trimming of the last three amino acids by Ras carboxyl CAAX endopeptidase I (RceI), and 2) methylation of the newly exposed prenylated cysteine residue by isoprenylcysteine carboxyl O-methyltransferase (Icmt) (Garcia-Mata et al., 2011). Isoprenylation of Cdc42-prenyl was experimentally confirmed by Maltese and Sheridan in cultured murine erythroleukemia (MEL) cells after radioactive labelling with [³H]-mevalonate (Maltese and Sheridan, 1990). Later on, cysteine residue 188 of Cdc42-prenyl was identified as prenylation site (Ziman et al., 1993). Same authors also demonstrated redistribution of Cdc42-prenyl to cytosolic fractions in a GGTaseI mutant strain in yeast, which draws the conclusion that Cdc42-prenyl is preferentially geranylgeranylated. This finding stays also true for the human Cdc42-prenyl, which prenylation was completely blocked by the inhibition of GGTaseI (Wilson et al., 1998). Interestingly, we and others reported that also the Cdc42-palm splice variant can undergo prenylation (Nishimura and Linder, 2013; Wirth et al., 2013). Our study and experiments by Nishimura and colleagues revealed that at least a small fraction of Cdc42-palm can bypass endopeptidase cleavage and carboxymethylation, resulting in a dually lipidated Cdc42-palm. In this case, cysteine residue 188 becomes geranylgeranylated, while cysteine residue 189 undergoes palmitoylation (Nishimura and Linder, 2013).

One additional observation of our study was that Cdc42-palm can be modified by both GGTaseI and FTase with similar efficiency and thus might accepts both C15 and C20 isoprenoids (Figure 1). A possible reason for different isoprenoid prevalence of Cdc42-prenyl and Cdc42-palm can be the differences in their C-terminal amino acid sequences. Indeed, composition of the *CaaX* box has been shown to be critically involved in the determination of the nature of prenylation reaction. C-terminal *CaaX* motif of Cdc42-prenyl contains CVLL amino acids, with leucine at the last position being responsible for its preferential prenylation with GGTaseI. In contrast, FTase can accept multiple amino acids at the last position of the *CaaX* motif (Moores et al., 1991). This can be a reason, why the C-terminal tail of Cdc42-palm (CCIF) is recognized by both GGTaseI and FTase.

## Cdc42 palmitoylation

Already in 1996, Marks and colleagues speculated that due to the differences in their C-terminal *CaaX*-motifs Cdc42-palm and Cdc42-prenyl can be differently lipidated (Marks and Kwiatkowski, 1996). This suggestion was experimentally confirmed only in 2008, when Kang and co-workers showed that Cdc42-palm is palmitoylated (Kang et al., 2008). The authors demonstrated palmitoylation of Cdc42-palm, both in cultured rat embryonic cortical neurons as well as in purified synaptosomal membrane fractions extracted from the whole adult rat brain. Moreover, using cultured cortical neurons as a model, they showed that palmitoylation of Cdc42-palm is a dynamic modification.

Nishimura and colleagues confirmed this finding and analyzed the lipid modification of the *CCaX*-box of Cdc42-palm in more detail. From their experiments, they concluded that Cdc42-palm gets first prenylated at cysteine residue 188 followed by palmitoylation at cysteine residue 189 (Nishimura and Linder, 2013). Nishimura and colleagues also demonstrated that upon inhibition of prenylation the palmitoylation was decreased too, suggesting that prenylation of Cys-188 might be necessary for subsequent palmitoylation of Cys-189 (Nishimura and Linder, 2013). At the same time, we studied palmitoylation of the brain-specific Cdc42-palm isoform and found that both cysteine residues, Cys-188 and Cys-189, can be palmitoylated (Wirth et al., 2013). In contrast to Nishimura’s data, we demonstrated that palmitoylation of Cdc42-palm was significantly increased in the presence of a GGTase inhibitor, pointing to the ability of Cys-188 to accept the modification with palmitate (Wirth et al., 2013). Dual lipid modifications by both prenylation and palmitoylation have previously been demonstrated for Rac1 as well as for several members of the Ras protein family, including N-Ras and H-Ras (Brunsveld et al., 2009). However, in all these proteins, the cysteine residues within the *CaaX*-motif are solely prenylated, while palmitoylated cysteine residues are localized upstream of the CaaX box. Moreover, the *aaX* sequence should undergo methylation-mediated cut before the protein can be palmitoylated. Therefore, Cdc42-palm represents the unique example with cysteine residue 188 modified by either prenylation or by palmitoylation, while the palmitoylated cysteine 189 resides within the untypical CaaX box (CCIF) and should thus bypass methylation excision (Figure 1).

Our finding that Cdc42-palm can undergo dual lipidation suggests the existence of two Cdc42-palm populations in the cell. The first one could consist of Cdc42-palm prenylated at cysteine 188 and palmitoylated at cysteine 189. In the second population, both cysteine residues might be palmitoylated. A prenylated population can arise by the initial modification of cysteine 188 either by GGTaseI or FTase. This will result in increased membrane affinity and thereby facilitate palmitoylation of cysteine 189 by specific palmitoyl acyl-transferases (PATs), which reside either in the Golgi compartment or at the plasma membrane (see below). Since the CCIF sequence of Cdc42-palm represents a non-canonical CaaX motif, a part of Cdc42-palm will be not recognized by prenylating enzymes, resulting in palmitoylation of both cysteine residues (i.e., Cys-188 and Cys-189). Since prenylation and palmitoylation possess different affinity for lipid bilayers (Roy et al., 2005) and can thus target Cdc42-palm to different membrane subdomains, the dual lipidation can represent a mechanism to regulate the intracellular distribution and functions of Cdc42-palm.

More recently, we performed a detailed analysis of palmitoylation stoichiometry for Cdc42-palm isoforms by applying acyl-PEGyl exchange gel shift (APEGS) assays (Kanadome et al., 2019). Using this assay, we unexpectedly found that i) only a relatively small fraction of Cdc42-palm isoform exists in palmitoylated form, and ii) Cdc42-palm overexpressed in N1E-115 cells as well as Cdc42-palm endogenously expressed in primary rat hippocampal neurons seem to be palmitoylated only at one cysteine residue. In the same study, we identified DHHC5 as a prominent Cdc42 PAT (Wirth et al., 2022). In addition, DHHC10 and DHHC17 can also be involved in Cdc42 palmitoylation. Interestingly, among these three Cdc42-palm specific PATs, only DHHC5 is residing at the plasma membrane (Brigidi et al., 2015). This might have an important implication for the regulation of Cdc42-palm functions at different intracellular compartments.

Although the molecular mechanisms of Cdc42-palm recognition by DHHC5 is not elucidated in detail, one possibility is the activation of DHHC5 *via* palmitoylation. Previously, it has been shown that DHHC5 could be palmitoylated at C-terminal cysteine residues outside of its catalytic core (Yang et al., 2010). This might result in conformational changes within the DHHC5 protein, leading to increased substrate specificity (Woodley and Collins, 2019). We have previously reported a reduced palmitoylation of Cdc42 in a mouse model of 22q11.2 deletion syndrome, in which DHHC8 is among the deleted genes (Moutin et al., 2017). Since overexpression of DHHC8 did not facilitate palmitoylation of Cdc42-palm (Wirth et al., 2022), DHHC8 could thus regulate enzymatic activity of DHHC5 towards Cdc42-palm *via* DHHC5 palmitoylation.

Moreover, palmitoylation of Cdc42-palm can be hierarchical organized in space and time. Indeed, Golgi-resided DHHC10 and/or DHHC17 can be responsible for the initial Cdc42 palmitoylation at Cys-188 immediately after synthesis. This will prevent prenylation and target Cdc42 to the plasma membrane. Our data revealed the increasing ratio of DHHC5 to Cdc42-palm in the brain during development, which could result in facilitation of Cdc42 palmitoylation within dendritic spines, which in turn will boost spinogenesis and spine maturation (see below).

Since palmitoylation is a dynamic and reversible process, Cdc42-palm can also undergo depalmitoylation. So far, only a few published works investigated the Cdc42 depalmitoylation. Cdc42-palm was shown to maintain asymmetric protein localization during cell division by coordinated interplay with catalytic active acyl protein thioesterase 1 (APT1) in breast cancer cells (MDA-MB-231) (Stypulkowski et al., 2018). The palmitoyl-protein thioesterases 1 (PPT1) was also found to be in favor of depalmitoylating Cdc42 (Gorenberg et al., 2022). However, the spatiotemporal palmitoylation/depalmitoylation pattern of Cdc42-palm remains elusive.

## Physiological role of Cdc42 lipidation: different lipids—different functions?

Cdc42 is highly conserved from yeast to mammals and plays an important role in the regulation of a plethora of cellular processes, including phagocytosis, cell polarization, chemotaxis, cell-migration and division (Etienne-Manneville and Hall, 2002; Cerione, 2004). Cdc42 is also critically involved in the organ and tissue homeostasis and has been associated with vascularization, immune system regulation, eye and skin development as well as cardiac and pancreatic organogenesis (for review see Melendez et al., 2011). Of note, all these organs ubiquitously express the Cdc42-prenyl isoform, while Cdc42-palm—so far—represents a brain specific isoform under physiological conditions. Therefore, the best-studied organ in terms of isoform-specific functions is the brain. It might be possible, however, that Cdc42-palm was overlooked in other tissues, since the majority of investigations published before the work by Kang and co-workers (Kang et al., 2008) did not discriminate between Cdc42 isoforms. Furthermore, in many publications describing Cdc42 knockdown, authors applied shRNAs targeting non-specific sites such as exon 2, which leads to the knockdown of both isoforms. On the other hand, starting from 2008, there is growing evidence demonstrating that Cdc42 isoforms are strictly distinguished in their functions, especially in neurons. Even though the possible mechanisms responsible for functional differences between both alternatively spliced variants are far from being completely understood, there are different hypothesis explaining how the proteins, differing only in their short C-terminal sequences, might possess different functions and thereby regulate distinct signaling pathways. One of them arises by the idea that palmitoylated proteins are enriched in membrane subdomains like lipid rafts, whereas isoporenylated proteins are excluded from these microdomains (Levental et al., 2010). Indeed, protein palmitoylation represents one of the best-characterized lipid raft targeting signals (Moffett et al., 2000; Zacharias et al., 2002). Detection of numerous signaling proteins within the lipid rafts led to the assumption that these structures represent a scaffolding platform or signaling hub, which boosts signal transduction by spatially recruiting signaling components and by preventing an inappropriate cross-talk between signaling pathways. This can also be true for Cdc42 isoforms, which specific lipidation (i.e., prenylation vs. palmitoylation) might contribute to a separated/different localization of both Cdc42 isoforms along the lipid membranes, leading to different downstream interaction partners and ultimately to initiation of different signaling cascades.

An important physiological function of Cdc42 is the activation of transcription factors such as NFκB, STAT3 and SRF (Perona et al., 1997; Wu et al., 2008). In particular, SRF-mediated transcription of many immediate early genes plays a crucial role for neuronal outgrowth and differentiation (Scandaglia et al., 2015). We have previously shown a higher specificity of Cdc42-palm towards activation of SRF-pathway in comparison with Cdc42-prenyl (Wirth et al., 2013). Noteworthy, such specificity is not mediated by changes in the intrinsic Cdc42 activation between isoforms, because expression of constitutively active mutants resulted in similar activation of SRF signaling. One possible explanation therefore could be a specific localization of Cdc42-palm in lipid rafts, which will result in its sustained interaction with raft-resided downstream-effectors such as Pak1, WASP or p38 leading to specific modulation of SRF-mediated signaling. This is supported by the observation that, although the mutation of palmitoylated Cys-189 does not affect proper localization of Cdc42-palm at the plasma membrane, it significantly reduced activation of SRF pathway to the level comparable with Cdc42-prenyl isoform (Wirth et al., 2013).

Kang and colleagues also investigated the functional consequences of the different lipid modifications of Cdc42 (Kang et al., 2008). Among others, they demonstrated that a constitutively active (CA) mutant of Cdc42-palm was more effective in inducing dendritic spine formation than Cdc42-prenyl CA mutant, and that the ability of Cdc42-palm CA to induce dendritic spines was abolished either by mutating the two C-terminal cysteine residues or by chemically inhibiting the palmitoylation by 2-bromopalmitate. More importantly, the authors showed that palmitoylation of Cdc42-palm isoform is dynamically regulated by neuronal activity. Upon glutamate treatment of cortical cultures, Cdc42-palm was very rapidly (i.e., within 5 min) de-palmitoylated and dislocated from dendritic spines. In contrast, triggering a seizure-like activity in the mouse brain by injection of kainic acid *in vivo* resulted in a significant increase of Cdc42 palmitoylation (Kang et al., 2008). Different intracellular expression patterns and, as consequence, functions of the Cdc42 splice variants were confirmed by Yap and co-authors. They found that Cdc42-palm (Cdc42E6) was critically involved in the formation of dendritic spines, whereas Cdc42-prenyl (Cdc42E7) was associated with axonogenesis (Yap et al., 2016). This observation was further confirmed by a study by Lee and colleagues, who found an enrichment of Cdc42-prenyl encoding mRNA in the axons of dorsal root ganglion neurons, where it was involved in regulating the increase in axon length. In contrast, Cdc42-palm mRNA was rather enriched in dendritic spines. Functional specificity of Cdc42 isoforms was demonstrated by the fact that global knock-down of CDC42 gene followed by rescue with Cdc42-palm was not able to facilitate an axonal outgrowth, while increased dendritic length (Lee et al., 2021).

On the other hand, our data suggest that both Cdc42 splice variants might be involved in the formation of dendritic protrusions and spines, since expression of either Cdc42-palm or Cdc42-prenyl in hippocampal neurons was able to rescue the inhibitory effect of the shRNA-mediated Cdc42 knock-down (Wirth et al., 2013). This is further supported by experiments in *Drosophila* demonstrating that fly Cdc42, which is homolog to mammalian Cdc42-prenyl, is required for multiple aspects of dendritic morphogenesis (Scott et al., 2003). The authors demonstrated that loss-of-function mutation of endogenous Cdc42 did not affect dendritic complexity but resulted in increased dendritic length accompanied by app. 50% reduction in dendritic spine density.

Detailed analysis of acylation-deficient mutants of Cdc42 revealed different roles of cysteine residues within Cdc42-palm C-terminus: If cysteine residue 188 was available as lipid acceptor, Cdc42-palm induced long dendritic protrusions only. Mutation of Cys-189 did not affect formation of long dendritic protrusions, while the number of short dendritic protrusion, which are thought to be spine precursors, was significantly reduced (Wirth et al., 2013). Together with the observation that Cdc42-palm undergoes palmitoylation in human brains, this suggests the importance of Cdc42 lipidation for the growth of new synapses as well as for activity-dependent structural and functional plasticity, including learning and long-term memory in human.

Another interesting aspect of lipid-specific regulation of Cdc42 functions is the finding that Cdc42-prenyl is essential for forming neuroprogenitor cells by activation of mTORC1 and thereby up-regulation of tissue-specific transcription factors, whereas Cdc42-palm drives the transition of neuroprogenitor cells into neurons by inhibiting mTORC1 signaling *via* activated Cdc42-associated kinase (ACK) (Endo et al., 2020). In the follow-up study, the authors identified the epidermal growth factor (EGF) receptor as an additional target of Cdc42-palm and ACK, which is down-regulated by their combined actions during neurogenesis. Thus, by down-regulating EGF receptor signaling, Cdc42-palm and ACK might determine the timing of terminal differentiation of neural progenitor cells into neurons. Mechanistically, Cdc42-palm and ACK-mediated EGF receptor degradation boost autophagy, which in turn protects neuronal progenitor from apoptosis and thus triggers their differentiation into neurons (Endo and Cerione, 2022). This idea is also in line with the accumulation of Cdc42-palm in the cortical plate, a place where neurons differentiate rather than proliferate (Olenik et al., 1999).

## Can Cdc42 lipidation be involved in pathological conditions?

Many *de novo* mutations within the CDC42 gene result in heterogeneous phenotypes with highly variable symptoms including facial dysmorphism, dysregulation of growth, haematological as well as immunological and neurodevelopmental abnormalities such as Takenouchi-Kosaki syndrome (TKS) (Martinelli et al., 2018; Su and Orange, 2020). However, there is only a restricted amount of data directly demonstrating pathological contribution of the Cdc42 lipidation.

In 2019, Gernez and colleagues reported about four patients carrying CDC42 gene mutations affecting the last three to five C-terminal amino acids of Cdc42-prenyl (i.e., part responsible for lipidation). These mutations were associated with severe auto-inflammatory symptoms including anemia, cytopenia, rashes, facial dysmorphisms and are associated with increased ferritin, C-reactive protein, and interleukin IL-18 levels. In addition, patients carrying these mutations were predisposed to the development of macrophage activation syndrome and hemophagocytic lymphohistiocytosis. Of note, the novel C-terminal variants in Cdc42 all occur within or in close spatial proximity to the C-terminal di-arginine motif implicated in membrane binding and lipidation of Cdc42-prenyl. One patient had a mutation within the CDC42 coding region leading to a replacement of the stop codon by a cysteine residue, resulting in a read-through and 24 additional amino acids (*192C*24). Another patient carried the R186C mutation. The other two patients carried a C188Y mutation, which unfortunately were not further characterized in detail (Gernez et al., 2019). Therefore, we can only speculate, that mutation of cysteine 188, which is particularly important for the post-translational modification and thus the spatial organization of Cdc42, might lead to mislocalization within cells and thus aberrant signaling.

Using whole exome sequencing, Bekhouche and co-authors detected a heterozygous mutation in a patient affecting only Cdc42-prenyl, resulting in the exchange of arginine in position 186 to a cysteine residue (R186C). The main clinical characteristics of this mutation include severe neonatal dermatitis accompanied by acute episodes of hepatomegaly with cytolysis, mild facial dysmorphy, and a permanent inflammatory syndrome with occasional monocytosis (Bekhouche et al., 2020). Detailed biochemical analysis revealed that R186C mutation leads to non-physiological palmitoylation at Cys-186 (Figure 2). This results in a misbalanced regulation of Cdc42-prenyl by the GDP dissociation inhibitor, GDI1, which specifically binds to the geranylgeranylated wild-type form of Cdc42-prenyl. Due to the artificial palmitoylation upstream of the isoprenylation site, Cdc42-prenyl-GDI1-interaction was impaired and the R186C mutant was abnormally anchored in the Golgi apparatus. One consequence of such aberrant localization was decreased actin filament polymerization: the patient carrying the R186C mutation contained around 30% less F-actin than normal. Moreover, authors reported a strong NF-κB hyper-activation, which relied on the palmitoylation-dependent Golgi apparatus retention of the Cdc42-prenyl R186C mutant. This in turn resulted in increased IL-8 and IL1β levels, which can explain the pathophysiology of the disease (Bekhouche et al., 2020).

Recently, Nishitani-Isa and colleagues further analyzed molecular details of autoinflammatory pathology evoked by R186C Cdc42-prenyl mutation. Using induced-pluripotent stem cells (iPSCs)-derived myeloid and macrophages cell lines established from the patients carrying this mutation, they reported that patient-derived cells secreted larger amounts of IL-1β in response to pyrin-activating stimuli (Nishitani-Isa et al., 2022). Noteworthy, pharmacological blockade of palmitoylation released R186C mutant from the Golgi apparatus and damped the IL-1β secretion to the wild-type levels. Similar results were also obtained for another patient mutation, *192C*24. This mutant was also aberrantly palmitoylated at the newly introduced Cys-192, leading to trapping at the Golgi apparatus and over-activation of the pyrin inflammasome (Figure 2). In contrast to results obtained by Bekhouche and co-authors, Nishitani-Isa and colleagues did not obtain any changes in GTPase activity of R186C mutant or its binding affinity towards GDIs, pointing out palmitoylation-mediated aberrant localization of Cdc42-prenyl in the Golgi apparatus as main driving force for pathological activation of the pyrin inflammasome.

These combined results not only demonstrate an unexpected association between aberrant palmitoylation of Cdc42-prenyl and activation of inflammatory responses, but also suggest that this pathway might represent a novel target for treatment of autoinflammatory diseases. Moreover, results of these studies suggest that although Cdc42-prenyl is ubiquitously expressed, pathological effects of its aberrant palmitoylation seem to be related to the Cdc42-prenyl population expressed outside of the brain tissue.
